# ﻿A review of *Terrilimosina* Roháček, 1983 (Diptera, Sphaeroceridae) from China with new records and three new species

**DOI:** 10.3897/zookeys.1241.146200

**Published:** 2025-06-12

**Authors:** Wenqiang Cao, Hui Dong, Ding Yang

**Affiliations:** 1 Department of Entomology, College of Plant Protection, China Agricultural University, 2 Yuanmingyuan West Road, Beijing 100193, China China Agricultural University Beijing China; 2 Key Laboratory of Southern Subtropical Plant Diversity, Fairylake Botanical Garden, Shenzhen & Chinese Academy of Sciences, Shenzhen 518004, China Fairylake Botanical Garden, Shenzhen & Chinese Academy of Sciences Shenzhen China

**Keywords:** China, key, new species, status changes

## Abstract

The 14 species of *Terrilimosina* Roháček now known from China are reviewed and keyed, including three new species (*T.bicruris***sp. nov.**, *T.dentata***sp. nov.**, and *T.digitata***sp. nov.**), three newly recorded species (*T.deemingi* Marshall, 1987, *T.longipexa* Marshall, 1987, and *T.unio* Marshall, 1987), and one subspecies (*T.maoershanensis* Su, 2011) is elevated to species rank. Chinese distribution records are updated, and figures and illustrations are provided for the 14 species of *Terrilimosina* recorded from China.

## ﻿Introduction

*Terrilimosina* species are distinguishable from other sphaerocerids by the posteriorly rounded discal cell (except *T.digitata* sp. nov.) (Fig. 29), broad alula, sinuate R_4+5_ and R_2+3_, costa extended beyond the tip of R_4+5_, surstylus with a comb-like row of bristles, and telescoping female postabdomen. Species of *Terrilimosina* are quite similar to each other in external characters, especially the female, but males can be distinguished by genitalia and S_5_, females by S_7_, T_7_, S_8_, T_8_, epiproct, and cercus, etc.

The genus *Terrilimosina* Roháček, 1983 includes 17 named species and subspecies (16 species according to Papp and Roháček 2021) from the Afrotropical, Nearctic, Oriental, and Palaearctic regions (Roháček et al. 2001; Marshall et al. 2011; Papp and Roháček 2021). Seven species and one subspecies were reported from China previously (Su and Liu 2009; Su et al. 2009; Su 2011; Dong et al. 2020). *Terrilimosinamaoershanensis* Su, 2011 was treated as a subspecies of *T.paralongipexa* Hayashi, 1992 (Su 2011). We here elevate it to species level after comparing these two species and further discuss the differences below under *T.maoershanensis*. Six further species from China, three of which are new to science, are added here based on the examination of 1611 specimens from Beijing, Chongqing, Fujian, Gansu, Guangxi, Guizhou, Hebei, Hubei, Jilin, Liaoning, Qinghai, Shaanxi, Shanxi, Tianjin, Tibet, Yunnan, and Zhejiang. Females of *T.parabrevipexa* Su and *T.parasmetanai* Su are here described for the first time. The 14 species of *Terrilimosina* now know from China are as follows: *T.bicruris* sp. nov., *T.brevipexa* Marshall, 1987, *T.capricornis* Su, 2009, *T.deemingi* Marshall, 1987, *T.dentata* sp. nov., *T.digitata* sp. nov., *T.longipexa* Marshall, 1987, *T.maoershanensis*, *T.nana* Hayashi, 1992, *T.parabrevipexa*, *T.paralongipexa*, *T.parasmetanai*, *T.schmitzi* (Duda, 1918), and *T.unio* Marshall, 1987.

## ﻿Materials and methods

Male and female abdomens were prepared by immersion in 15% sodium hydroxide (NaOH) at room temperature for 6–8 hours before being rinsed in distilled water and transferred to glycerol. Illustrations of genitalia were made using an OLYMPUS SZ61 microscope and then shaded in Adobe Photoshop 2020 (Adobe Services, California USA). The specimens and cleared abdomens were stored in a microcentrifuge tube with glycerol and preserved in the vials of alcohol with the rest of the specimen after examination.

Habitus photographs were taken using a Canon 7D Mark II digital camera and a Canon MP-E 65 mm macro lens, with multiple images stacked with Helicon Focus v. 5.3 (Helicon Soft Ltd., Kharkiv, Ukraine) and assembled using Adobe Photoshop 2020. Measurements were obtained using a calibrated micrometer.

**Figure 1.**
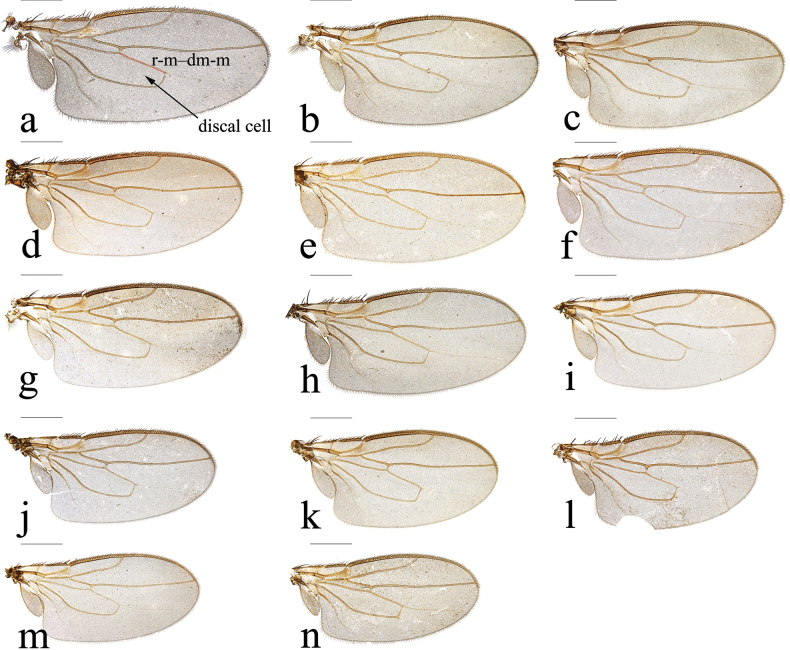
*Terrilimosina* wings. **a.***T.bicruris* sp. nov.; **b.***T.dentata* sp. nov.; **c.***T.parasmetanai* Su & Liu; **d.***T.brevipexa* Marshall; **e.***T.parabrevipexa* Su & Liu; **f.***T.maoershanensis* Su; **g.***T.schmitzi* (Duda); **h.***T.deemingi* Marshall; **i.***T.capricornis* Su & Liu; **j.***T.longipexa* Marshall; **k.***T.paralongipexa* Hayashi; **l.***T.nana* Hayashi; **m.***T.unio* Marshall; **n.***T.digitata* sp. nov. Scale bar: 0.5 mm.

Most terminology for genital structures follows Marshall (1987) except the term ‘postgonite’ is used here instead of ‘paramere’. Chaetotaxy is described using the general term ‘setae’ for macrotrichia, with larger and typically named setae referred to as bristles and very small setae as setulae. Stout, short, straight setae are referred to as spines and the term “spur” is applied to the generally curved stout ventral preapical seta of the hind tibia. The distance of *r-m*–*dm-m* is shown as Fig. 1a.

All specimens are deposited in the Entomological Museum of China Agricultural University (**EMCAU**).

### ﻿Key to males of the species of *Terrilimosina* found in China

**Table:** 

1	Fore coxa, trochanter, and at least basal half of femur yellow (Figs 6, 8, 12a)	**2**
–	Fore coxa, trochanter, and femur brownish to dark brown (Figs 4, 28)	**4**
2	Syntergite 1+2 with a weakly sclerotized anteromedial patch (Fig. 12b); sternite 5 with triangular, densely setulose posteromedial process (Fig. 13d); surstylus with dense posterior setulae, digital anteroventral projection, and a stout tooth-like bristle at posterior corner (Fig. 13a, b)	***T.digitata* sp. nov.**
–	Syntergite 1+2 evenly sclerotized; sternite 5 with rectangular, densely setulose posteromedial process or patch of posteromedial setulae (Figs 7d, 9d); surstylus bare posteriorly, anteroventral part with an anterior process, posterior corner without a tooth-like bristle but sometimes with a posteroventral process (Figs 7b, 9b)	**3**
3	Sternite 5 with patch of posteromedial setulae (Fig. 7d); surstylus short, with anteroventral process (with long anterior process and a dense comb-like row of bristles at apex), and a triangular posteroventral process (Fig. 7b)	***T.capricornis* Su**
–	Sternite 5 with densely setulose posteromedial process (Fig. 9d); surstylus long, with anteroventral process (with short anterior process and a sparse comb-like row of bristles at apex), and unmodified posteroventral corner (Fig. 9b)	***T.deemingi* Marshall**
4	Syntergite 1+2 with a weakly sclerotized anteromedial patch (Fig. 12b)	***T.unio* Marshall**
–	Syntergite 1+2 well sclerotized (Fig. 2b)	**5**
5	Surstylus with 1–5 very short, blunt bristles (not typical comb-like row of bristles) at apex of posteroventral process (Figs 5b, 19b, 21b)	**6**
–	Surstylus with a comb-like row of bristles on inner ventral surface (Fig. 6)	**8**
6	Sternite 5 with posteromedial patch of posteromedial setulae (Fig. 5d); surstylus with ~ 5 very short, blunt bristles at apex of posteroventral lobe (Fig. 5b)	***T.brevipexa Marshall***
–	Sternite 5 with two patches of stout posterior bristles (Fig. 19d) or a rectangular posteromedial process (Fig. 21d); surstylus with 1 or 2 short, blunt bristles at apex of posteroventral lobe (Figs 19b, 21b)	**7**
7	Sternite 5 with two patches of short, stout posterior bristles (Fig. 19d); sternite 6 with a small posteromedial process (Fig. 19d); surstylus with 2 short, blunt bristles at apex of posteroventral lobe (Fig. 19b); postgonite slender, with sharp apex (Fig. 19c)	***T.nana* Hayashi**
–	Sternite 5 with a rectangular posteromedial process with dense setulae (Fig. 21d); sternite 6 unmodified; surstylus with 1 very small stout bristle at apex of posteroventral lobe (Fig. 21b); postgonite broad, with blunt apex (Fig. 21c)	***T.parabrevipexa* Su**
8	Sternite 5 with shallow posteromedial notch (Figs 11d, 27d)	**9**
–	Sternite 5 with distinct posteromedial lobe or process	**10**
9	Sternite 5 with a row of thin posterior bristles and sparse setulae (Fig. 27d); surstylus with a straight comb-like row of bristles internally (Fig. 27a, b); postgonite with a broad preapical process internally and blunt apex (Fig. 27c); distiphallus with a small sharp posteroventral process medially (Fig. 27c)	***T.schmitzi* (Duda)**
–	Sternite 5 with 8 robust tooth-like bristles and patch of dense posteromedial setulae (Fig. 11d); surstylus with a bent comb-like row of bristles externally (Fig. 11); postgonite with sharp apex; distiphallus unmodified ventrally (Fig. 11c)	***T.dentata* sp. nov.**
10	Surstylus with anterior process and without an internal lobe (Figs 3a, b, 25b)	**11**
–	Surstylus without anterior process and with an anterior lobe internally (Figs 15a, b, 17a, b, 23a, b)	**12**
11	Sternite 5 with a small posteromedial process (with dense setulae) (Fig. 25d); surstylus with a very small comb-like row of bristles ventrally (Fig. 25b); postgonite with broad anterior process and shallowly bifurcated apex (Fig. 25c)	***T.parasmetanai* Su**
–	Sternite 5 with 3 posterior processes (with dense setulae and middle one apically bifurcate) (Fig. 3d); surstylus with a stout comb-like row of bristles internally (Fig. 3a, b); postgonite with an inner posterior lobe (with a small process medially) and a shallow preapical notch (Fig. 3c)	***T.bicruris* sp. nov.**
12	Sternite 5 with dense long bristles posteriorly and a basally constricted posteromedial lobe (with ~ 5 short robust marginal bristles and tufts of small, flat setulae) (Fig. 15d); surstylus with 5 robust tooth-like bristles ventrally and 1 longer robust tooth-like bristle posteriorly (Fig. 15a, b)	***T.longipexa* Marshall**
–	Sternite 5 with sparse short bristles posteriorly and a basally constricted posteromedial lobe (with tufts of small, flat setulae) (Figs 17d, 23c, d); surstylus with 1 robust tooth-like bristle posteriorly (Figs 17a, b, 23a, b)	**13**
13	Sternite 5 with a posteromedial lobe shallowly constricted basally (with tufts of small, flat setulae and a narrow lateral process) and a pair of bristles at the base of posteromedial lobe (Fig. 23c, d); surstylus with 4 robust bristles at the internal lobe and compact comb-like row of bristles internally (Fig. 23a, b)	***T.paralongipexa* Hayashi**
–	Sternite 5 with a posteromedial lobe deeply constricted basally (with tufts of small, flat setulae and a broad lateral process) and two pairs of bristles at the base of the posteromedial lobe (Fig. 17d); surstylus with 1 robust bristle at the internal lobe and incompact comb-like row of bristles internally (Fig. 17a, b)	***T.maoershanensis* Su**

### ﻿Generic description

Head generally dull; four interfrontal bristles, anterior one shorter; two orbital bristles; orbital setulae forming a long row between interfrontal bristles and eye. Eye oval. One long anterior gena bristle. Antenna dark brown, postpedicel darker; arista shortly ciliate.

Postpronotal lobe with two bristles, internal one shorter than anterior dorsocentral bristle; two postsutural dorsocentral bristles, anterior one 2 × as long as dorsocentral setula in front of it; ~ 8 rows of irregular acrostichal setulae in front of suture; prescutellar medial pair of acrostichal setulae nearly as long as anterior dorsocentral bristle. Scutellum subtriangular, with two pairs of scutellar bristles. Two katepisternal bristles, anterior one minute. Legs brown. Wing infuscate, pale brownish; costa extended beyond R_4+5_; R_4+5_ sinuate, almost straight apically; discal cell long, posteriorly rounded, without pigmented process of M_4_ extending beyond dm-m. Alula large with round apex. Haltere with pale brown knob and stem.

## ﻿Species descriptions

### ﻿ Terrilimosina bicruris sp. nov.

Taxon classificationAnimaliaDipteraSphaeroceridae

EFABC124-E366-551C-A140-A9E9A2D60DD6

https://zoobank.org/24AE05E6-06DD-4D37-8DFE-334345F299DB

Figs 1a , 2 , 3

#### Type locality.

China, Xizang: Linzhi, Mt. Sejila, 3745m, 6.vii.2018, Qicheng Yang.

#### Type material.

***Holotype***: China: - **Xizang**: • ♂; Linzhi, Mt. Sejila; 3745 m, 6.vii.2018; Qicheng Yang; in alcohol. (EMCAU).

#### Diagnosis.

**Figure 2.**
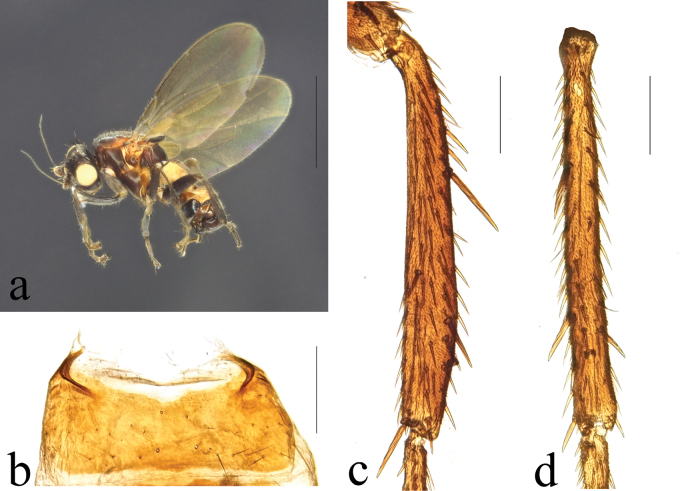
*Terrilimosinabicruris* sp. nov. male habitus. **a.** Lateral; **b.** Syntergite 1+2 dorsal; **c.** left mid tibia anterior; **d.** Left mid tibia dorsal. Scale bars: 1.0 mm (**a, b**); 0.5 mm (**c, d**).

**Figure 3.**
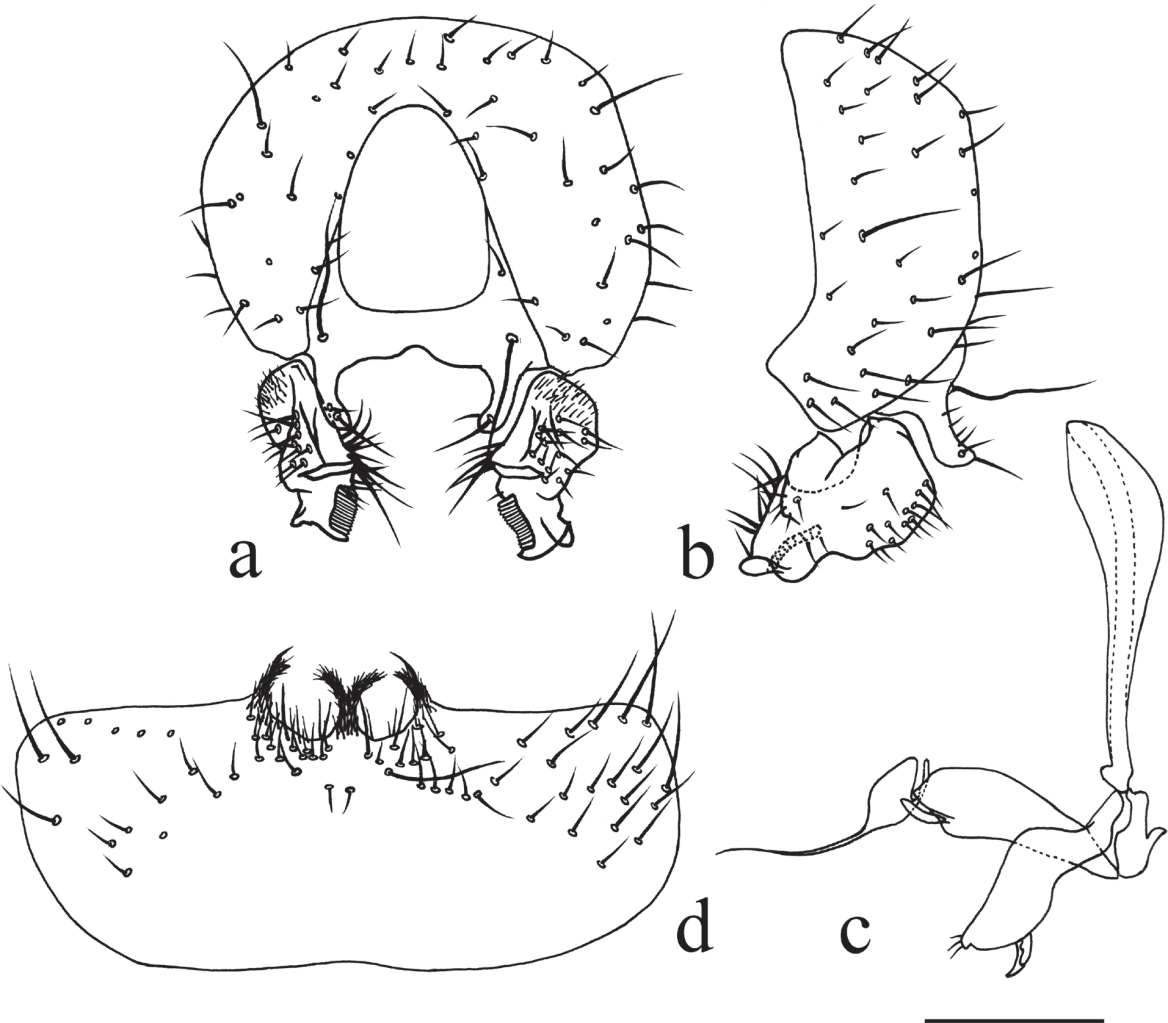
*Terrilimosinabicruris* sp. nov. structures of male postabdomen. **a.** Terminalia posterior; **b.** Terminalia lateral; **c.** Phallus and associated structures lateral; **d.** S_5_ ventral. Scale bar: 0.1 mm.

Arista ~ 5.6 × as long as postpedicel. Hind tibia ventrally with one thin apical bristle; hind basitarsomere apically with one short anterodorsal bristle. Syntergite 1+2 well sclerotized (Fig. 2b). S_5_ with sparse setulae and three densely setulose posterior process (middle one bifurcated) (Fig. 3d).

#### Description.

**Male** (Fig. 2a–d). Body length 1.7 mm, wing 1.8 mm. General color black.

Eye height 2.5 × genal height at point of maximum eye height. One long vibrissa. Arista ~ 5.6 × as long as postpedicel. Postgonite with an inner posterior lobe (with a small process medially), a shallow notch preapically and ~ 3 small apical setulae (Fig. 3c).

Mid tibial chaetotaxy as in Fig. 2c, d, ventrally with one short anteroventral bristle below middle and one longer apical bristle; hind tibia ventrally with one thin apical bristle; hind basitarsomere apically with one short anterodorsal bristle. Wing (Fig. 1a) infuscate, pale brownish; C-index = 0.9; *r-m*–*dm-m:dm-m* = 3.5.

Tergites and sternites sparsely and shortly setulose. Syntergite 1+2 well sclerotized (Fig. 2b). Sternite 5 with sparse setulae and three densely setulose posterior process (middle one bifurcated) (Fig. 3d).

Male genitalia: Epandrium (Fig. 3a, b) sparsely and shortly setose, with a longer dorsolateral bristle. Cercus long and fingerlike, with a long bristle basally and a setae apically (Fig. 3a, b). Surstylus with a stout comb-like row of ventral bristles internally and some small anterior and posteroventral setae (Fig. 3a, b). Basiphallus small, with a small posterior process (Fig. 3c). Postgonite with an inner posterior lobe (with a small process medially), a shallow notch preapically and ~ 3 small apical setulae (Fig. 3c). Distiphallus with a ventral process linking basal and apical parts (with a thin posteromedial process, two broad posterolateral processes and a very long but thin anterior process (Fig. 3c).

**Female.** Unknown.

#### Etymology.

The specific name is derived from the Latin *bicruris* (bifurcated), and refers to the bifurcated posterior process on the S_5_.

#### Distribution.

China (Xizang).

#### Comments.

*Terrilimosinabicruris* sp. nov. resembles *T.parasmetanai* Su in having a distiphallus with a long apical process, but differs in having S_5_ with three densely setulose posterior processes and middle one bifurcated (Fig. 3d), long fingerlike cercus, surstylus with a stout comb-like row of ventral bristles internally and postgonite with an inner posterior lobe (with a small process medially) (Fig. 3a–c). *Terrilimosinaparasmetanai* has a small densely setulose posteromedial process on S_5_ (Fig. 25d), short triangular cercus, a very small comb-like row of surstylar bristles ventrally and a shallowly bifurcated postgonite apex (Fig. 25a–c).

### ﻿ Terrilimosina brevipexa

Taxon classificationAnimaliaDipteraSphaeroceridae

Marshall, 1987

62C9C2B1-EB4E-521C-B8BE-E2AE20A88AD1

Figs 1d , 4 , 5

Terrilimosina brevipexa Marshall, 1987: 503. - Roháček et al. 2001: 272 [World Catalog of Sphaeroceridae]; Su and Liu 2009: 51–52 [illustr.]; Su 2011:116–117, 214 [redescription, illustr.]; Dong et al. 2020: 430 [Species Catalog of China].

#### Material examined.

China: - **Jilin**: • 1 ♂, 1 ♀; Wangqing, Zhongxianglinchang; 558 m, 7.vii.2023; Yishen Xiao; (all EMCAU).

#### Diagnosis.

Male S_5_ convex and densely setulose posteromedially; these setulae enlarged and flattened at apex of posteromedial lobe (Marshall 1987: fig. 5). Surstylus laterally setulose at base; ventrally with anterior and posterior lobes, posterior lobe with an internal comb of short, stout bristles at apex (Marshall 1987: figs 1, 2).

**Figure 4.**
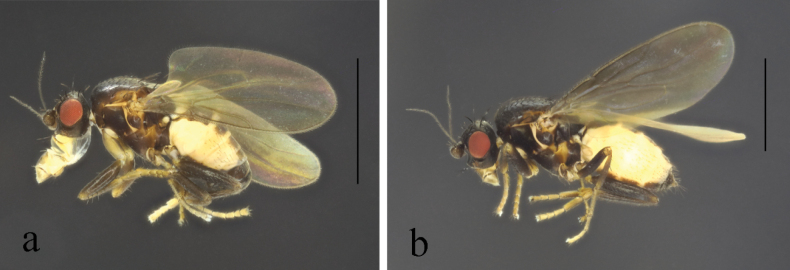
*T.brevipexa* Marshall, 1987 habitus. **a.** Male lateral; **b.** Female lateral. Scale bars: 1.0 mm.

**Figure 5.**
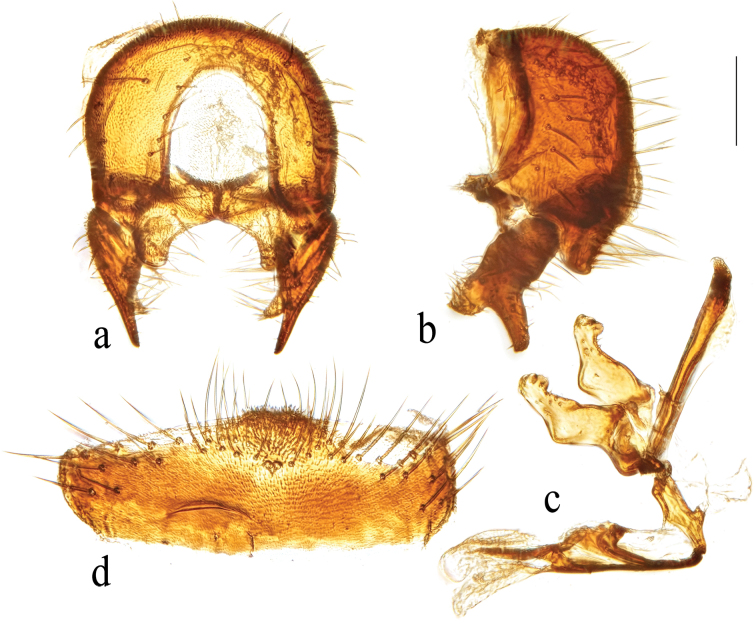
*T.brevipexa* Marshall, 1987 structures of male postabdomen. **a.** Terminalia posterior; **b.** Terminalia lateral; **c.** Phallus and associated structures lateral; **d.** S_5_ ventral. Scale bar: 0.1 mm.

#### Type locality.

Japan, Shikoku, Ishizuchi Mt. National Park, Tsuchigoya [1400 m].

#### Distribution.

China (Jilin); Japan (Shikoku).

#### Comments.

Chinese records of this species are from Jilin only (Su and Liu 2009; Su 2011; Dong et al. 2020).

### ﻿ Terrilimosina capricornis

Taxon classificationAnimaliaDipteraSphaeroceridae

Su & Liu, 2009

94DEFBDC-39E2-573B-945F-4080E278AC1A

Figs 1i , 6 , 7

Terrilimosina capricornis Su & Liu in Su et al. 2009: 808. - Su 2011: 115–116, 212 [redescription, illustr.]; Dong et al. 2016: 81 [illustr.]; Dong et al. 2020: 431 [Species Catalog of China].

#### Material examined.

China: - **Chongqing**: • 4 ♂; Wuxixian, Yintiaoling, Guanshanmaizitang; 2168 m, 17.viii.2022; Xulong Chen. - **Fujian**: • 1 ♀; Wuyishan National Park, Tongmu, Gaoqiao; 609 m, 24.viii.2023; Xiaoyan Liu; (L). - **Guizhou**: • 1 ♂, 1 ♀; Suiyang, Kuangkuoshuizhongxinzhan; 3.vi.2010; Yan Li. - **Hubei**: • 2 ♂, 3 ♀; Shennongjia, Qianjiaping; 1707 m, 8.vi.2023; Jiuzhou Liu. - **Shaanxi**: • 3 ♂, 10 ♀; Ningshan, Huodigou; 2034 m, 14.viii.2022; Bing Zhang. - **Xizang**: • 1 ♀; Motuo, Damuxiang; 1530 m, 24.vi.2018; Qicheng Yang. - **Yunnan**: • 1 ♂; Lushui, Fengxueyakou; 3105 m, 13.viii.2023; Qicheng Yang. - **Zhejiang**: • 1 ♀; Tianmushan, Qianmutian; 30.vii.2011; Tingting Zhang. (all EMCAU).

#### Diagnosis.

Sternite 5 with patch of posteromedial setulae and long setae at the posterior edge (Fig. 7d) Surstylus with an anteroventral process (with long anterior process above and a dense comb-like row of bristles at apex) and a triangular posteroventral process (Fig. 7a–c).

**Figure 6.**
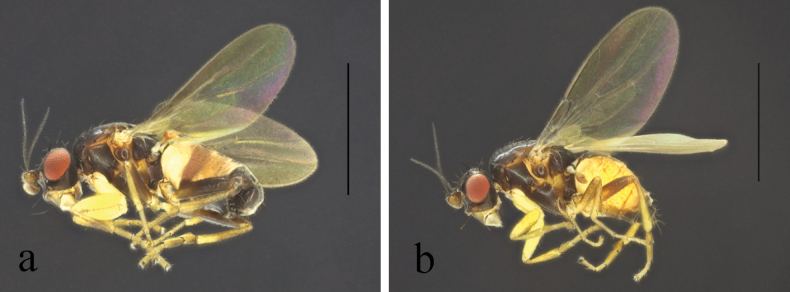
*T.capricornis* Su & Liu, 2009 habitus. **a.** Male lateral; **b.** Female lateral. Scale bars: 1.0 mm.

**Figure 7.**
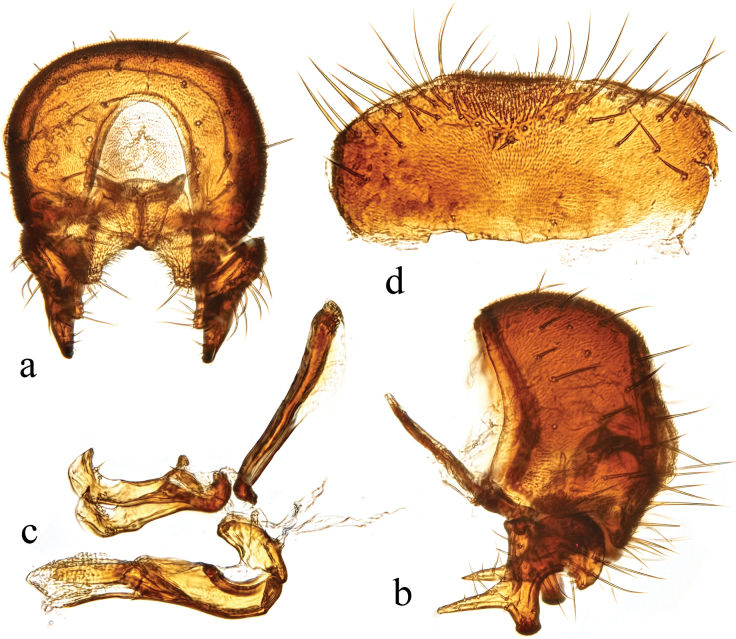
*T.capricornis* Su & Liu, 2009 structures of male postabdomen. **a.** Terminalia posterior; **b.** Terminalia lateral; **c.** Phallus and associated structures lateral; **d.** S_5_ ventral. Scale bar: 0.1 mm.

#### Type locality.

China, Guangxi Zhuang Autonomous Region, Maoer Mountain [1800 m].

#### Distribution.

China (Chongqing, Fujian, Guangxi, Guizhou, Hubei, Shaanxi, Xizang, Yunnan, Zhejiang).

#### Comments.

Previous Chinese records of this species are from Guangxi, Jiangxi, and Zhejiang (Su 2011; Dong et al. 2016, 2020). Here we newly record it from Chongqing, Fujian, Guizhou, Hubei, Shaanxi, Tibet, and Yunnan. Dong et al. (2020) recorded authorities of this species which are different from the authors of the publication.

### ﻿ Terrilimosina deemingi

Taxon classificationAnimaliaDipteraSphaeroceridae

Marshall, 1987

86C9F5D7-17F3-59EA-89DA-9E1CC8E1EF50

Figs 1h , 8 , 9

Terrilimosina deemingi Marshall, 1987: 504. - Roháček et al. 2001: 272 [World Catalog of Sphaeroceridae].

#### Material examined.

China: - **Hebei**: • 1 ♂; Chengde, Wulingshan, Liushuigou; 17.vii.2016; Wenmin Xiao; (L). - **Xizang**: • 1 ♀; Motuo, Mt. Nanzelama; 1930 m, 23.vi.2018; Liang Wang. - **Yunnan**: • 7 ♂, 7 ♀; Lushui, Pianmazhenlushuiyakou; 3105 m, 12.viii.2023; Qicheng Yang. (all EMCAU).

#### Diagnosis.

Sternite 5 with a densely setulose posteromedial process and short setae (Fig. 9d). Surstylus with an anteroventral process (with short anterior process and a sparse comb-like row of bristles at apex) and unmodified posteroventral corner (Fig. 9a–c).

**Figure 8.**
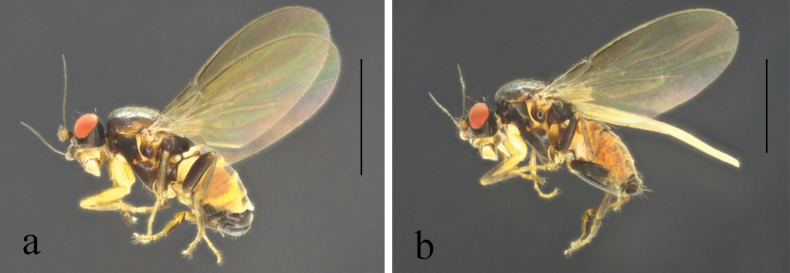
*T.deemingi* Marshall, 1987 habitus. **a.** Male lateral; **b.** Female lateral. Scale bars: 1.0 mm.

**Figure 9.**
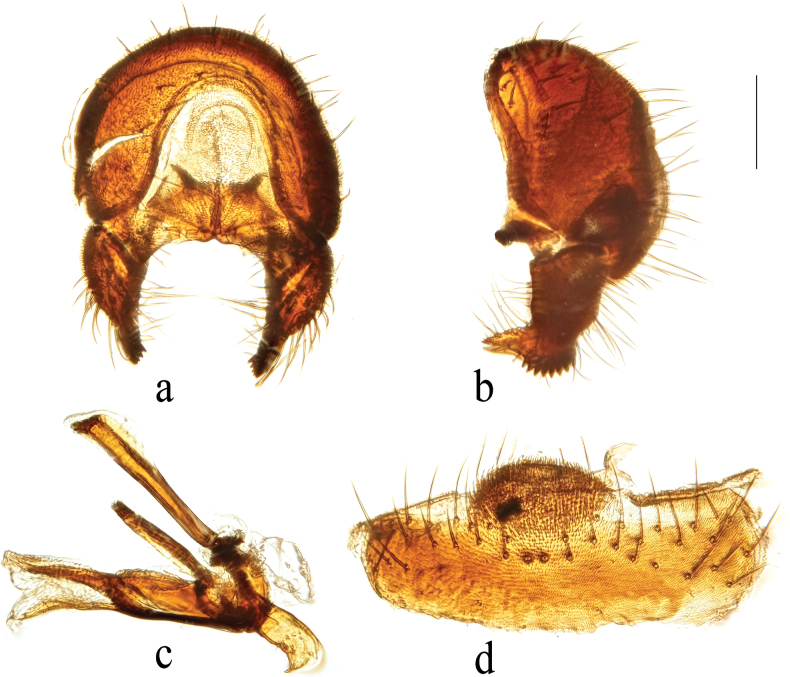
*T.deemingi* Marshall, 1987 structures of male postabdomen. **a.** Terminalia posterior; **b.** Terminalia lateral; **c.** Phallus and associated structures lateral; **d.** S_5_ ventral. Scale bar: 0.1 mm.

#### Type locality.

Nepal, between Ghopte and Thare Pati [3200 m].

#### Distribution.

China (Hebei, Xizang, Yunnan); Nepal.

#### Comments.

*Terrilimosinadeemingi* Marshall, newly recorded from China, resembles *T.capricornis* Su, from which it differs in having a densely setulose posteromedial process and short setae on S_5_ (Fig. 9d), an anteroventral process (with short anterior process and a sparse comb-like row of bristles at apex), and unmodified posteroventral corner on the surstylus (Fig. 9a–c). *Terrilimosinacapricornis* Su has patch of posteromedial setulae and long setae on S_5_ (Fig. 7d), an anteroventral process (with long anterior process and a dense comb-like row of bristles at apex) and a triangular posteroventral process on the surstylus (Fig. 7a–c).

### ﻿ Terrilimosina dentata sp. nov.

Taxon classificationAnimaliaDipteraSphaeroceridae

8FEED279-FB42-5E9B-8853-9ED031A4A88C

https://zoobank.org/F894778F-4B1E-441A-9E8F-14B755194C2A

Figs 1b , 10 , 11

#### Type locality.

China, Xizang: Yadong, Zhongyinkou’an, 3610 m, 14.vii.2018, Liang Wang.

#### Type material.

***Holotype***: China: - **Xizang**: • ♂; Yadong, Zhongyinkou’an; 3610 m, 14.vii.2018; Liang Wang; in alcohol. (EMCAU).

#### Diagnosis.

Arista ~ 5.7 × as long as postpedicel. Hind basitarsomere with one thin anteroventral bristle basally, one slightly flattened anterodorsal bristle apically and one very small ventral spine subapically. Syntergite 1+2 well sclerotized. Sternite 5 with sparse posterior setae, eight robust tooth-like bristles at middle and patch of dense posteromedial setulae (Fig. 11d). Postgonite sinuate, with sharp apex and ~ 3 setae at middle (Fig. 11c).

**Figure 10.**
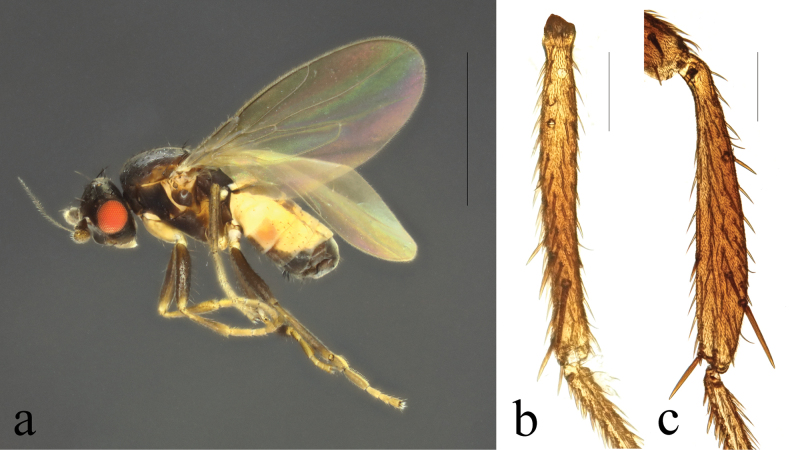
*Terrilimosinadentata* sp. nov. male habitus. **a.** Lateral; **b.** Left mid tibia dorsal; **c.** Left mid tibia anterior. Scale bars: 1.0 mm (**a**); 0.1 mm (**b, c**).

**Figure 11.**
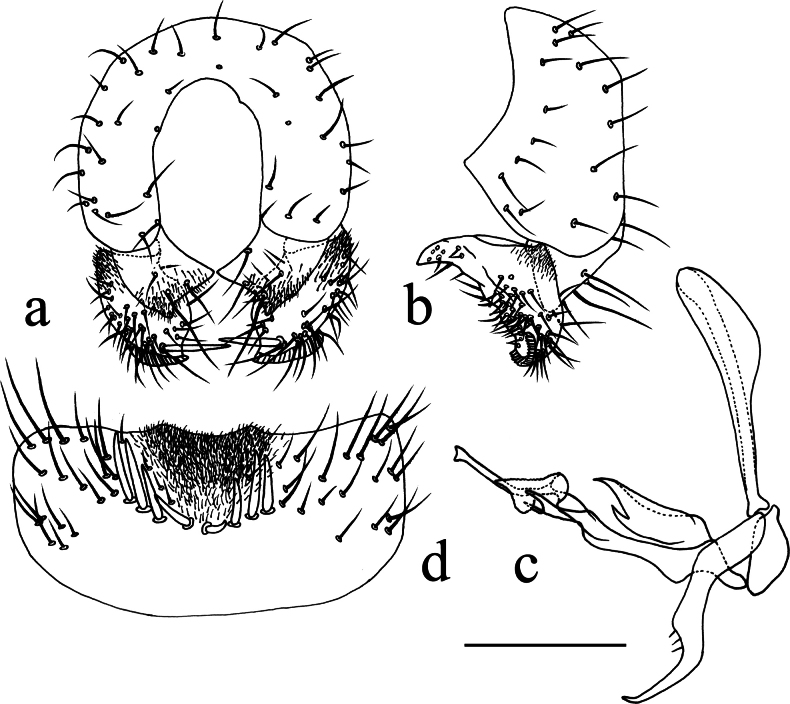
*Terrilimosinadentata* sp. nov. structures of male postabdomen. **a.** Terminalia posterior; **b.** Terminalia lateral; **c.** Phallus and associated structures lateral; **d.** S_5_ ventral. Scale bar: 0.1 mm.

#### Description.

**Male** (Fig. 10a). Body length 1.7 mm, wing 1.8 mm. General color brown.

Eye height 2.0 × genal height at point of maximum eye height. One very long vibrissa. Arista ~ 5.7 × as long as postpedicel.

Thorax black. Mid tibial chaetotaxy as in Fig. 10b, c, ventrally with one very short anteroventral bristle below middle and one longer apical bristle; hind basitarsomere with one thin anteroventral bristle basally, one slightly flattened anterodorsal bristle apically and one very small ventral spine subapically. Wing (Fig. 1b) infuscate, pale brownish; C-index = 1.0; *r-m*–*dm-m:dm-m* = 3.8.

Tergites and sternites sparsely and shortly setulose. Syntergite 1+2 well sclerotized. Sternite 5 with sparse posterior setae, eight robust tooth-like spines at middle and patch of dense posteromedial setulae (Fig. 11d).

Male genitalia. Epandrium (Fig. 11a, b) sparsely and shortly setulose. Cercus broad triangular, with a long bristle subapically, some short setae and dense setulae apically (Fig. 11a, b). Surstylus with sharp anterior process, a curved comb-like row of bristles surrounded by dense setae, dense setulae posterodorsally and a long inner-posterior tooth-like spine (Fig. 11a, b). Basiphallus short, with round posterior margin (Fig. 11c). Postgonite sinuate, with sharp apex and ~ 3 setae at middle (Fig. 11c). Distiphallus with a weakly sclerotized and apically sharp dorsal part covering a well sclerotized ventral part with a ventral process and ear-like dorsal process, apex with a long and weakly sclerotized process basally covered by membranous sheet with serrate margins (Fig. 11c).

**Female.** Unknown.

#### Etymology.

The specific name is derived from the Latin *dentatus* (dentate), and refers to the robust spines on the male S_5_.

#### Distribution.

China (Shanxi, Xizang, Yunnan).

#### Comments.

*Terrilimosinadentata* sp. nov. resembles *T.nana* Hayashi, from which it differs in having the male S_5_ with eight robust tooth-like spines medially and a patch of dense posteromedial setulae (Fig. 11d), surstylus with a curved comb-like row of bristles surrounded by dense setae and a long inner-posterior tooth-like spine (Fig. 11a, b). *Terrilimosinanana* Hayashi has two patches of short, stout posterior bristles on the male S_5_ (Fig. 19d) and two short, blunt bristles on the apex of the posteroventral lobe of the surstylus (Fig. 19a, b).

### ﻿ Terrilimosina digitata sp. nov.

Taxon classificationAnimaliaDipteraSphaeroceridae

4D639924-7B17-544D-97C4-7878467040B3

https://zoobank.org/BE1CB2CD-CFA8-406C-BA9B-8747C3F33DB9

Figs 1n , 12 , 13

#### Type locality.

China: Xizang: Motuo, Mt. Nanzelama, 1930 m, 23.vi.2018, Liang Wang.

#### Type material.

***Holotype***: China: - **Xizang**: • ♂; Motuo, Mt. Nanzelama; 1930 m, 23.vi.2018; Liang Wang; in alcohol. (EMCAU). ***Paratype***: • 1 ♂; Same data as holotype. (EMCAU).

#### Diagnosis.

**Figure 12.**
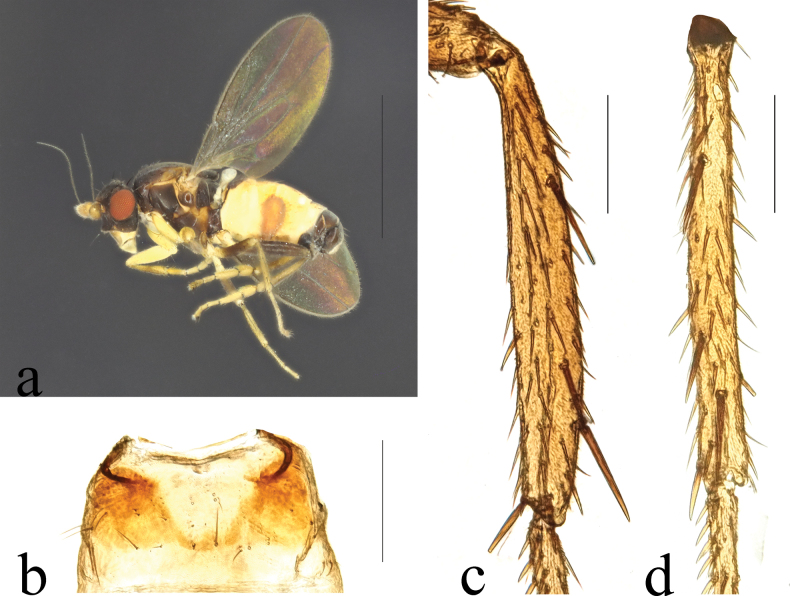
*Terrilimosinadigitata* sp. nov. habitus. **a.** Male lateral; **b.** Syntergite 1+2 dorsal; **c.** Left mid tibia anterior; **d.** Left mid tibia dorsal. Scale bars: 1.0 mm (**a, b**); 0.1 mm (**c, d**).

**Figure 13.**
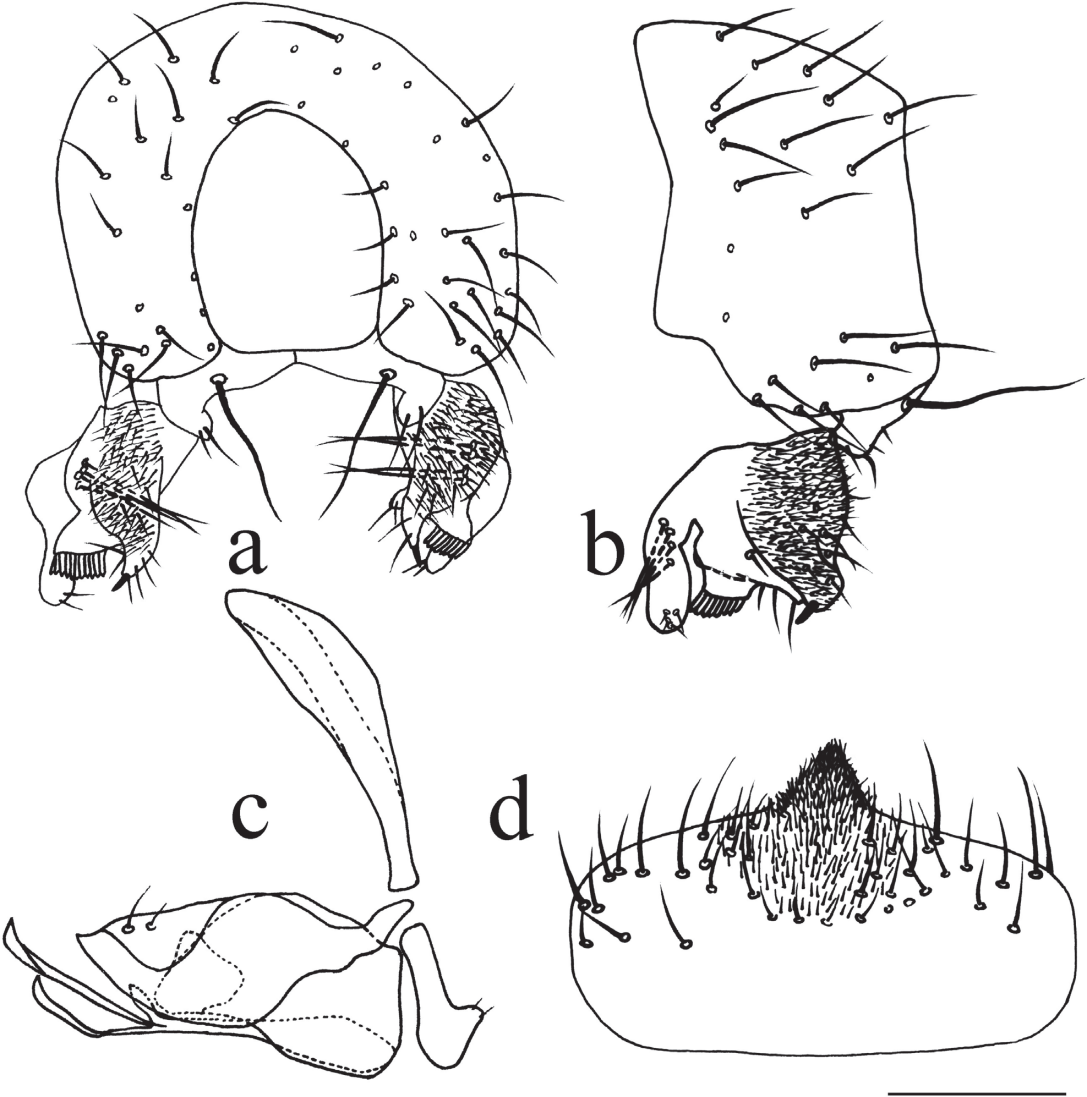
*Terrilimosinadigitata* sp. nov. structures of male postabdomen. **a.** Terminalia posterior; **b.** Terminalia lateral; **c.** Phallus and associated structures lateral; **d.** S_5_ ventral. Scale bar: 0.1 mm.

Arista ~ 5.1–5.3 × as long as postpedicel. Mid and hind legs pale brown, femur (except base) darker; fore legs yellow. Syntergite 1+2 with a weakly sclerotized anteromedial patch (Fig. 12b). Sternite 5 with triangular, densely setulose posteromedial process (Fig. 13d). Surstylus with a digital anteroventral projection dorsally (with patch of long bristles internally).

#### Description.

**Male** (Fig. 12a). Body length 1.6–1.8 mm, wing 1.4–1.6 mm. General color dark brown.

Eye height 2.2–2.3 × genal height at point of maximum eye height. One long vibrissa. Antenna pale brown, postpedicel darker; arista ~ 5.1–5.3 × as long as postpedicel.

Mid and hind legs pale brown, femur (except base) darker; fore legs yellow. Mid tibial chaetotaxy as in Fig. 12c, d, ventrally with one very short anteroventral bristle below middle and one longer apical bristle; hind basitarsomere with one short anteroventral bristle basally, one short anterodorsal bristle apically and one short robust ventral spine subapically. Wing (Fig. 1n) infuscate, pale brownish; C-index = 1.1–1.2; *r-m*–*dm-m:dm-m* = 3.3–3.5. Halter with pale brown knob and paler stem.

Abdomen pale brown. Tergites and sternites sparsely and shortly setulose; tergites small and weakly sclerotized. Syntergite 1+2 with a weakly sclerotized anteromedial patch (Fig. 12b). Sternite 5 with triangular, densely setulose posteromedial process (Fig. 13d).

Male genitalia: Epandrium (Fig. 13a, b) sparsely and shortly setulose. Cercus short, with rounded apex, a long bristle basally (Fig. 13a, b). Surstylus with dense posterior setulae, a digital anteroventral projection dorsally (with patch of long bristles internally), a short anteroventral process ventrally (with a short comb-like row of bristles apically) and a stout tooth-like spine at posterior corner (Fig. 13a, b). Basiphallus short, with blunt posterior process (Fig. 13c). Postgonite widened from middle to apex, with a weakly sclerotized patch subapically (with 2 small setulae dorsally) (Fig. 13c). Distiphallus with a broad dorsal part (with a small sclerite apically) covering slender ventral part with laterodorsal process subapically and sharp apex linking with two long membranous process (Fig. 13c).

**Female.** Unknown.

#### Etymology.

The specific name is derived from the Latin *digitatus* (digital), and refers to the digital anteroventral projection on the surstylus.

#### Distribution.

China (Xizang).

#### Comments.

*Terrilimosinadigitata* sp. nov. resembles *T.parasmetanai* Su, from which it differs in having syntergite 1+2 with a weakly sclerotized anteromedial patch (Fig. 12b), surstylus with a digital anteroventral projection dorsally (with patch of long bristles internally) and a stout tooth-like spine on the posterior corner and the postgonite with a weakly sclerotized dorsal patch (Fig. 13a–c). *Terrilimosinaparasmetanai* Su has a well sclerotized anteromedial syntergite 1+2, surstylus with a sharp anterior process and a well sclerotized postgonite (Fig. 25a–c).

### ﻿ Terrilimosina longipexa

Taxon classificationAnimaliaDipteraSphaeroceridae

Marshall, 1987

06C68059-FBD1-555A-BB0D-BDC41445EDFA

Figs 1j , 14 , 15

Terrilimosina longipexa Marshall, 1987: 505. - Roháček et al. 2001: 272 [World Catalog of Sphaeroceridae].

#### Material examined.

China: - **Chongqing**: • 1 ♂; Beibei, Jinyunshan; 772 m, 11.vi.2021; Bing Zhang. - **Gansu**: • 2 ♀; Kangxian, Yangbameiyuangou; 998 m, 27.viii.2023; Liang Wang. - **Guangxi**: • 1 ♂; Huapingzhen, Huangjingdongtiankeng; 950 m, 18.iv.2024; Wenqiang Cao. - **Hubei**: • 1 ♀; Yichang, Xingshan County, Longmenhe Village; 1446 m, 10.vi.2023; Xingnong Xu. - **Xizang**: • 1 ♂; Chayuxiancheng; 2015 m, 13.viii.2020; Qicheng Yang. - **Yunnan**: • 1 ♂, 2 ♀; Yiliang, Xiaocaoba, 1761 m; 17.vii.2023, Sihan Li. (all EMCAU).

#### Diagnosis.

Sternite 5 posteriorly with dense long bristles, a basally deep constricted posteromedial lobe (with ~ 5 short robust marginal bristles and tufts of small, flat setulae), and two pairs of bristles (posterior pair thinner) at the base of the posteromedial lobe of S_5_ (Fig. 15d). Surstylus with three robust bristles on the internal lobe of the surstylus, five robust ventral tooth-like spines, and one longer robust tooth-like posterior spine (Fig. 15a–c).

**Figure 14.**
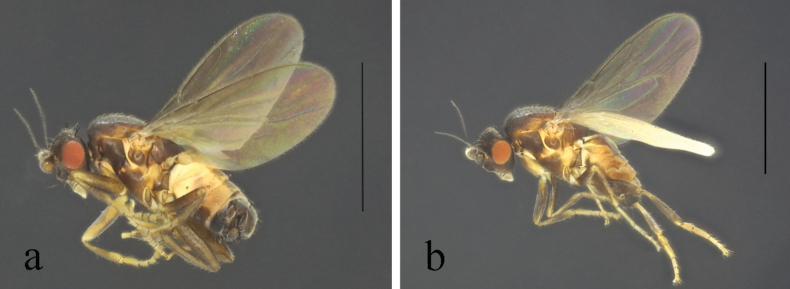
*T.longipexa* Marshall, 1987 habitus. **a.** Male lateral; **b.** Female lateral. Scale bars: 1.0 mm.

**Figure 15.**
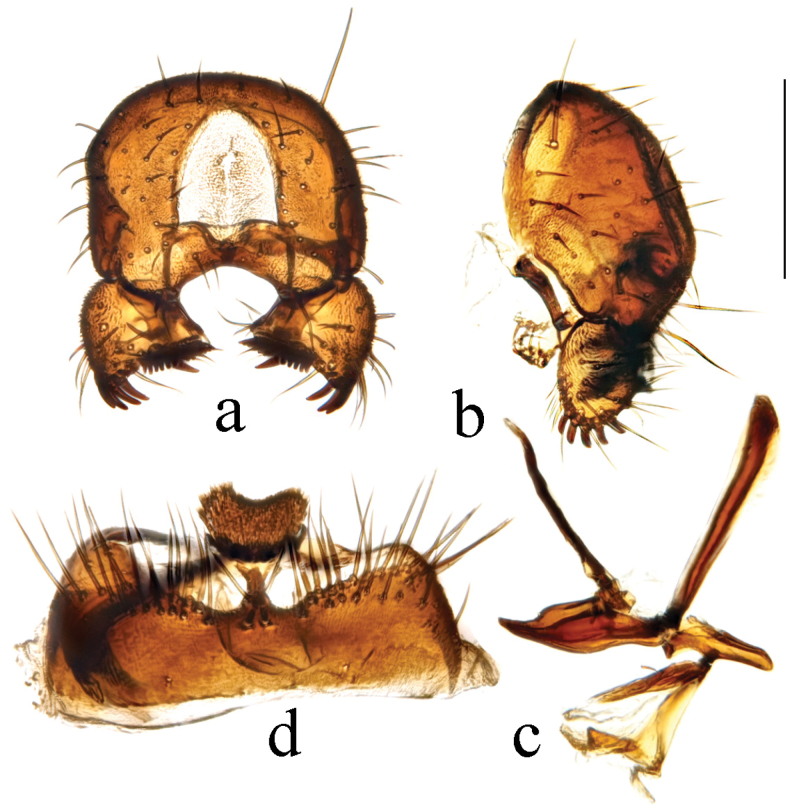
*T.longipexa* Marshall, 1987 structures of male postabdomen. **a.** Terminalia posterior; **b.** Terminalia lateral; **c.** Phallus and associated structures lateral; **d.** S_5_ and S_6_ ventral. Scale bar: 0.1 mm.

#### Type locality.

Japan, Shikoku, Ishizuchi Mt. National Park, Omogo Valley [700 m].

#### Distribution.

China (Chongqing, Gansu, Guangxi, Hubei, Xizang, Yunnan); India (Uttaranchal); Japan (Shikoku); Nepal.

#### Comments.

*Terrilimosinalongipexa* Marshall, newly recorded from China, resembles *T.paralongipexa* Hayashi, from which it differs in having dense long bristles on S_5_, a basally deep constricted posteromedial lobe (with ~ 5 short robust marginal bristles and tufts of small, flat setulae) on S_5,_ two pairs of bristles (posterior pair thinner) at the base of the posteromedial lobe of S_5_ (Fig. 15d), three robust bristles on the internal lobe of the surstylus, and five robust ventral tooth-like spines and one longer robust tooth-like posterior spine on the surstylus (Fig. 15a–c). *Terrilimosinaparalongipexa* Hayashi has sparser short setae on S_5_, a basally shallow constricted posteromedial lobe (with tufts of small, flat setulae and a narrow lateral process) on S_5,_ a pair of bristles at the base of the posteromedial lobe of S_5_ (Fig. 23c, d), four robust bristles on the internal lobe of the surstylus, some ventral setae, and one shorter robust tooth-like posterior bristle on the surstylus (Fig. 23a, b).

### ﻿ Terrilimosina maoershanensis

Taxon classificationAnimaliaDipteraSphaeroceridae

Su, 2011 stat. nov.

ECCB8930-4714-54B5-B3E4-AE19D24ACA8A

Figs 1f , 16 , 17

Terrilimosina paralongipexa maoershanensis Su, 2011: 120–121, 217. - Dong et al. 2020: 431 [Species Catalog of China].

#### Material examined.

China: - **Chongqing**: • 2 ♂; Beibei, Jinyunshan; 772 m, 11.vi.2021; Bing Zhang; • 1 ♂; Jinyunshan; 772 m, 10.vi.2021; Hang Zhou. - **Gansu**: • 1 ♂; Kangxian, Yangbameiyuangou; 998 m, 27.viii.2023; Liang Wang. - **Guizhou**: • 1 ♂; Suiyang, Kuangkuoshuibaohuquzhongxinzhan; 5.vi.2010; Dan Zhou. - **Hubei**: • 1 ♂; Shennongjia Forest District, Xiaoqianjiaping; 1719 m, 8.vi.2023; Xingnong Xu. - **Xizang**: • 1 ♂; Motuo, 80K; 2125 m, 20.viii.2020; Qicheng Yang. - **Yunnan**: • 1 ♂; Yiliang, Xiaocaoba; 1992 m, 14.vii.2023; Sihan Li. (all EMCAU).

#### Diagnosis.

Sternite 5 with sparse setae, a narrow posteromedial lobe (with tufts of small, flat setulae and a broad lateral process), and two pairs of bristles at the base of posteromedial lobe of S_5_ (Fig. 17d). Surstylus with one robust bristle and an incompact comb-like row of bristles internally (Fig. 17a–c).

**Figure 16.**
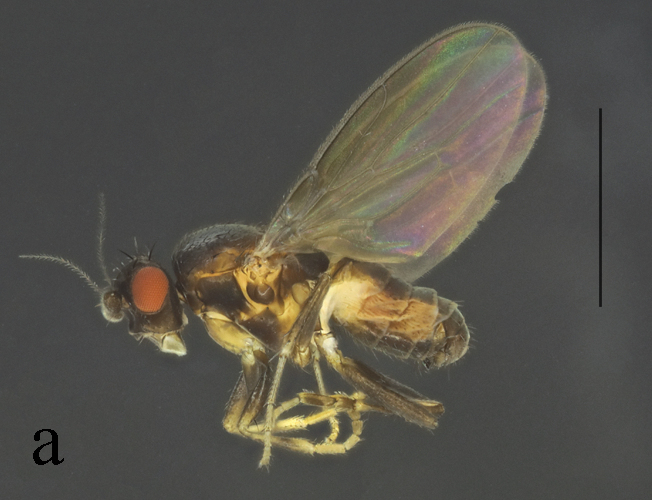
*T.maoershanensis* Su, 2011 male habitus. **a.** Lateral. Scale bar: 1.0 mm.

**Figure 17.**
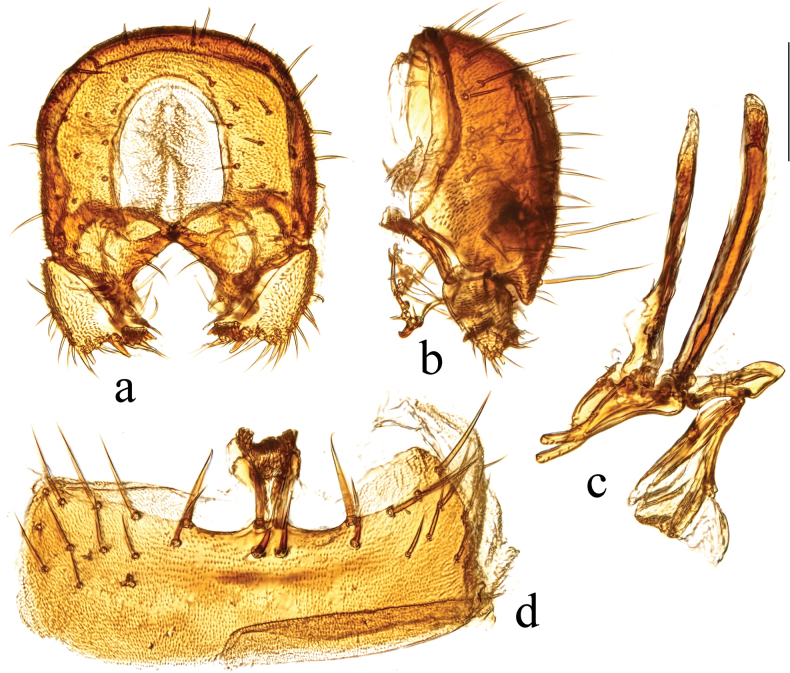
*T.maoershanensis* Su, 2011 structures of male postabdomen. **a.** Terminalia posterior; **b.** Terminalia lateral; **c.** Phallus and associated structures lateral; **d.** S_5_ ventral. Scale bar: 0.1 mm.

#### Type locality.

China, Guangxi, Maoershan [1800 m].

#### Distribution.

China (Chongqing, Gansu, Guangxi, Guizhou, Hubei, Xizang, Yunnan).

#### Comments.

Previous Chinese records of this species are from Guangxi (Su 2011; Dong et al. 2020). Here we present new records from Chongqing, Guizhou, Gansu, Hubei, Tibet, and Yunnan.

*Terrilimosinamaoershanensis* Su resembles *T.paralongipexa* Hayashi, from which males differ in having sparse setae on S_5_, a narrow posteromedial lobe (with tufts of small, flat setulae and a broad lateral process) on S_5,_ two pairs of bristles at the base of posteromedial lobe of S_5_ (Fig. 17d), one robust bristles and an incompact comb-like row of bristles on internal surface of the surstylus (Fig. 17a–c). *Terrilimosinaparalongipexa* Hayashi has denser setae on S_5_, a broad posteromedial lobe (with tufts of small, flat setulae and a narrow lateral process) on S_5,_ a pair of bristles at the base of posteromedial lobe of the S_5_ (Fig. 23c, d), and four robust bristles and a compact comb-like row of bristles on the internal surface of the surstylus (Fig. 23a, b). Because of these distinct differences in the male terminalia, we here treat *T.maoershanensis* as a distinct species from *T.paralongipexa*, and elevate it to the species level.

### ﻿ Terrilimosina nana

Taxon classificationAnimaliaDipteraSphaeroceridae

Hayashi, 1992

FD9AABA6-28CC-55E8-9CBB-A43760D8BEF2

Figs 1l , 18 , 19

Terrilimosina nana Hayashi, 1992: 569.- Roháček et al. 2001: 272 [World Catalog of Sphaeroceridae]; Su et al. 2009: 807–808 [redescription, illustr.]; Su 2011: 117–118, 215 [redescription, illustr.]; Dong et al. 2020: 431 [Species Catalog of China].

#### Material examined.

China: - **Beijing**: • 1 ♂, 1 ♀; Miyunxian, Huayuancun; 672 m, 3.vi.2018; Chunmin Zhang. - **Gansu**: • 1 ♂; Linxia Hui Autonomous Prefecture, Hezheng County, Songmingyan Scenic Area; 2456 m, 15.viii.2023; Ding Yang. - **Hebei**: • 1 ♂; Wulingshan, Zhonggusi; 12.vi.2019; Ding Yang. - **Hubei**: • 1 ♂; Yichang, Longmenhecun; 1261 m, 9.vi.2023; Jiuzhou Liu. - **Liaoning**: • 1 ♂; Fushun, Lijiacun; 540 m, 20.vi.2020; Tao Li; (M). - **Shaanxi**: • 1 ♂; Ningshan, Shibazhangpubu; 1116 m, 14.viii.2022; Bing Zhang. - **Tianjin**: • 1 ♂; Jizhou, Baxianshan; 221–706 m, 28.vi.2019; Dongyue He. (all EMCAU).

#### Diagnosis.

Male S_5_ with two patches of short, stout posterior bristles (Fig. 19d). Male S_6_ with a small posteromedial process (Fig. 19d). Surstylus with two short, blunt bristles on the apex of the posteroventral lobe (Fig. 19a, b).

**Figure 18.**
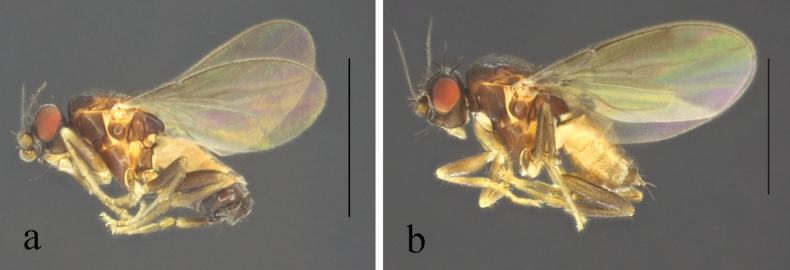
*T.nana* Hayashi, 1992 habitus. **a.** Male lateral; **b.** Female lateral. Scale bars: 1.0 mm.

**Figure 19.**
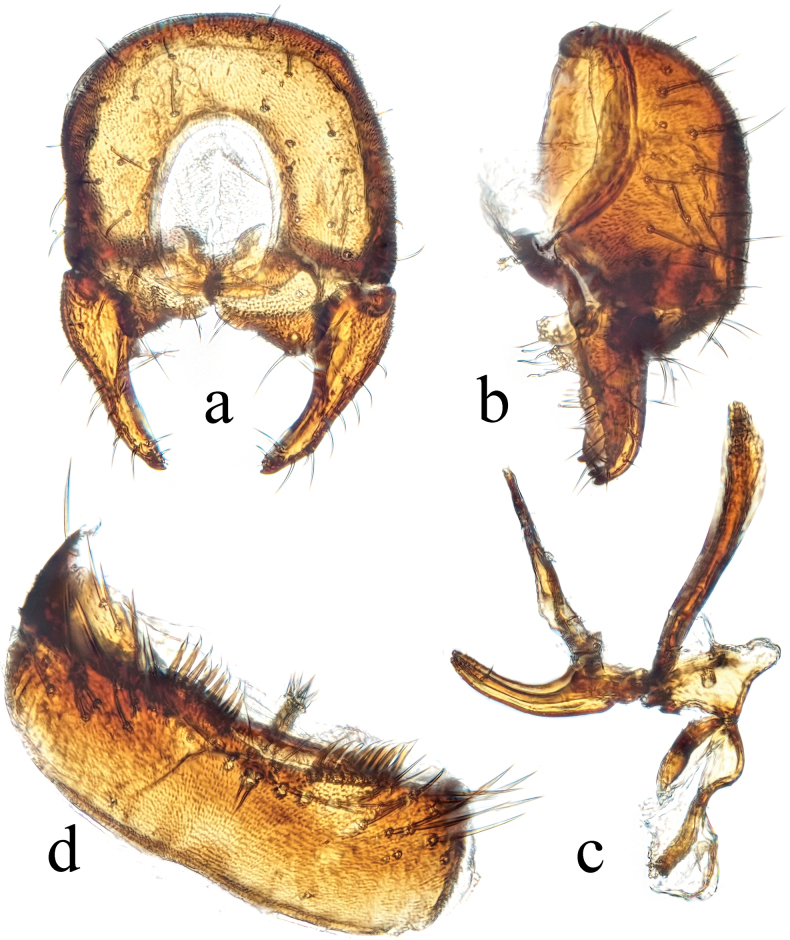
*T.nana* Hayashi, 1992 structures of male postabdomen. **a.** Terminalia posterior; **b.** Terminalia lateral; **c.** Phallus and associated structures lateral; **d.** S_5_ and S_6_ ventral. Scale bar: 0.1 mm.

#### Type locality.

Japan, Saitama, Iruma-gun, Moroyama.

#### Distribution.

China (Beijing, Gansu, Hebei, Hubei, Liaoning, Shaanxi, Tianjin); Japan (Saitama).

#### Comments.

Previous Chinese records of this species are from Liaoning (Su et al. 2009; Su 2011; Dong et al. 2020). Here we present new records from Beijing, Gansu, Hebei, Hubei, Shaanxi, and Tianjin.

### ﻿ Terrilimosina parabrevipexa

Taxon classificationAnimaliaDipteraSphaeroceridae

Su & Dong, 2009

B9502DA6-8F05-599B-B5D6-6854B3C6C412

Figs 1e , 20 , 21

Terrilimosina parabrevipexa Su & Dong in Su and Liu 2009: 53. - Su 2011: 118–120, 216 [redescription, illustr.]; Dong et al. 2020: 431 [Species Catalog of China].

#### Material examined.

China: - **Chongqing**: • 1 ♂; Beibei, Jinyunshan; 772 m, 10.vi.,2021; Bing Zhang; • 1 ♀; Jinyunshan; 772 m, 10.vi.2021; Hang Zhou. - **Gansu**: • 1 ♂; Kangxian, Pingheba; 1625 m, 28.viii.2023; Liang Wang. - **Guizhou**: • 1 ♀; Suiyang, Kuangkuoshuizhongxinzhan; 2.vi.2010; Dan Zhou. - **Hebei**: • 1 ♀; Chengde, Wulingshan, Zhufeng; 2085 m, 17.vii.2016; Wenmin Xiao. - **Hubei**: • 4 ♂, 4 ♀; Shennongjia, Qianjiaping; 1707 m, 8.vi.2023; Jiuzhou Liu. - **Jilin**: • 1 ♀, Helong, Wolongguanhuzhan; 737 m, 6.vii.2023; Yishen Xiao. - **Shanxi**: • 1 ♂, 1 ♀; Lvliang, Pangquangou Badaogou,; 1930 m, 15.vii.2023; Leyou Zhang. - **Shaanxi**: • 27 ♂, 16 ♀; Ningshan, Huodigou; 2034 m, 14.viii.2022; Bing Zhang. (all EMCAU).

#### Brief diagnosis of female.

The female (Fig. 21e, f) of this species is newly associated. T_7_ rectangular, with two pairs long posterior setae and some small setae. T_8_ wide, divided into three parts (middle one small and lateral part extended ventrally). Epiproct with a weakly sclerotized anterior notch and pair of setae. Cercus long, with long and sinuate setae. Spermathecae (2+1) cylindrical, constricted medially, with narrow apical invagination and tiny spine outside.

**Figure 20.**
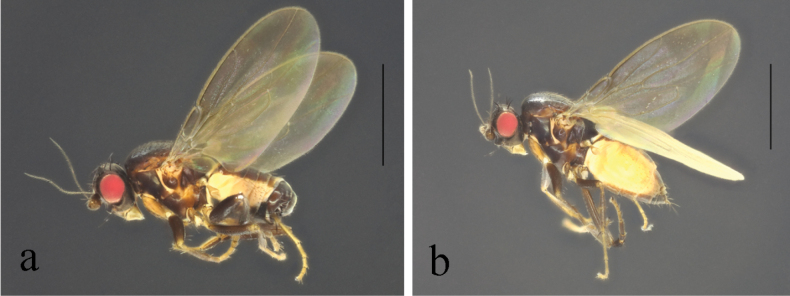
*T.parabrevipexa* Su & Dong, 2009 habitus. **a.** Male lateral; **b.** Female lateral. Scale bars: 1.0 mm.

**Figure 21.**
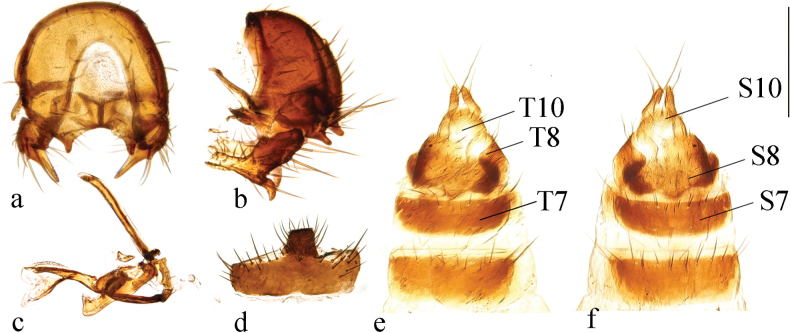
*T.parabrevipexa* Su & Dong, 2009. **a–d.** Structures of male postabdomen: **a.** Terminalia posterior; **b.** Terminalia lateral; **c.** Phallus and associated structures lateral; **d.** S_5_ ventral; **e, f.** Structures of female postabdomen: **e.** Dorsal; **f.** Ventral. Scale bar: 0.1 mm.

#### Diagnosis.

Male S_5_ with a rectangular posteromedial process (Fig. 21d). Surstylus with an anteroventral and posteroventral process (Fig. 21c).

#### Type locality.

China, Ningxia Province, Mt. Liu-p’an.

#### Distribution.

China (Chongqing, Gansu, Guizhou, Hebei, Hubei, Jilin, Ningxia, Shanxi, Shaanxi).

#### Comments.

Previous Chinese records of this species are from Ningxia and Shaanxi (Su and Liu 2009; Su 2011; Dong et al. 2020). Here we present new records from Chongqing, Gansu, Guizhou, Hebei, Hubei, and Shanxi. Dong et al. (2020) recorded authorities of this species which are different from the authors of the publication. The authors of this species in the original paper is different from the publication authors (Su and Liu 2009).

Females of *T.parabrevipexa* resemble *T.brevipexa* Marshall, from which they differ in having an intact T_7_, a shallow anterior process on wide S_8_ (width more than 2 × length) and narrow weakly sclerotized anterior notch on epiproct (Fig. 21e, f). *Terrilimosinabrevipexa* Marshall has a shallow posterior concavity on T_7_, intact S_8_ (width ~ 1.5 × length) and broad weakly sclerotized anterior notch on hypoproct (Marshall 1987: figs 6–8).

### ﻿ Terrilimosina paralongipexa

Taxon classificationAnimaliaDipteraSphaeroceridae

Hayashi, 1992

4B1322C7-96B9-582F-98F0-EC373FD12FC3

Figs 1k , 22 , 23

Terrilimosina paralongipexa Hayashi, 1992: 572. - Roháček et al. 2001: 272 [World Catalog of Sphaeroceridae]; Su and Liu 2009: 52 [illustr.].
Terrilimosina paralongipexa paralongipexa . - Su 2011: 121–122, 218 [redescription, illustr.]; Dong et al. 2020: 431 [Species Catalog of China].

#### Material examined.

China: - **Chongqing**: • 1 ♀; Liangping, Dahebashuiku; 457 m, 10–11.viii.2012; Zhifei Li. - **Gansu**: • 1 ♀; Kangxian, Yangbameiyuangou; 998 m, 27.viii.2023; Liang Wang. - **Guizhou**: • 1 ♂; Yanhexian, Mayangziranbaohuqu; 1.x.2007; Zaihua Yang; • 2 ♂, 2 ♀; Mayanghelantinghe; 3.x.2007; Xiangsi Huo. - **Hubei**: • 5 ♂, 3 ♀; Shennongjia, Muyu; 1235 m, 6.vi.2023; Jiuzhou Liu. - **Jilin**: • 1 ♂, 1 ♀; Wangqing, Cilaoyagou; 500 m, 3.vii.2023; Yuetian Gao. - **Liaoning**: • 1 ♀; Fushun, Lijiacun; 540 m, 8.vii.2020; Tao Li; (M). - **Xizang**: • 4 ♀; Motuo, 80K; 2125 m, 20.viii.2020; Qicheng Yang. - **Yunnan**: • 1 ♀; Yiliang, Xiaocaoba; 1992 m, 14.vii.2023; Sihan Li. (all EMCAU).

#### Diagnosis.

Male S_5_ with sparse short setae, a basally shallow constricted posteromedial lobe (with tufts of small, flat setulae and a narrow lateral process), and a pair of bristles at the base of the posteromedial lobe of S_5_ (Fig. 23d). Surstylus with four robust bristles on the internal lobe of the surstylus, numerous ventral setae, and one short robust tooth-like posterior bristle (Fig. 23a, b).

**Figure 22.**
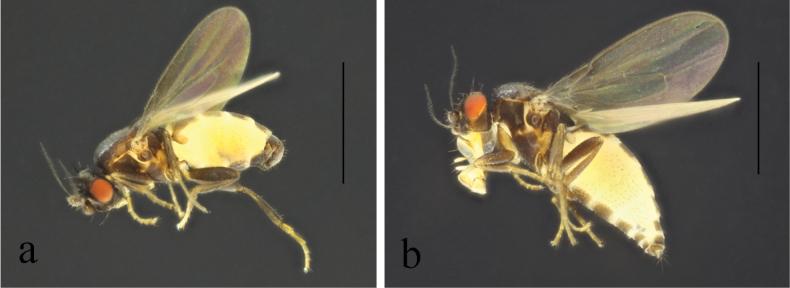
*T.paralongipexa* Hayashi, 1992 habitus. **a.** Male lateral; **b.** Female lateral. Scale bars: 1.0 mm.

**Figure 23.**
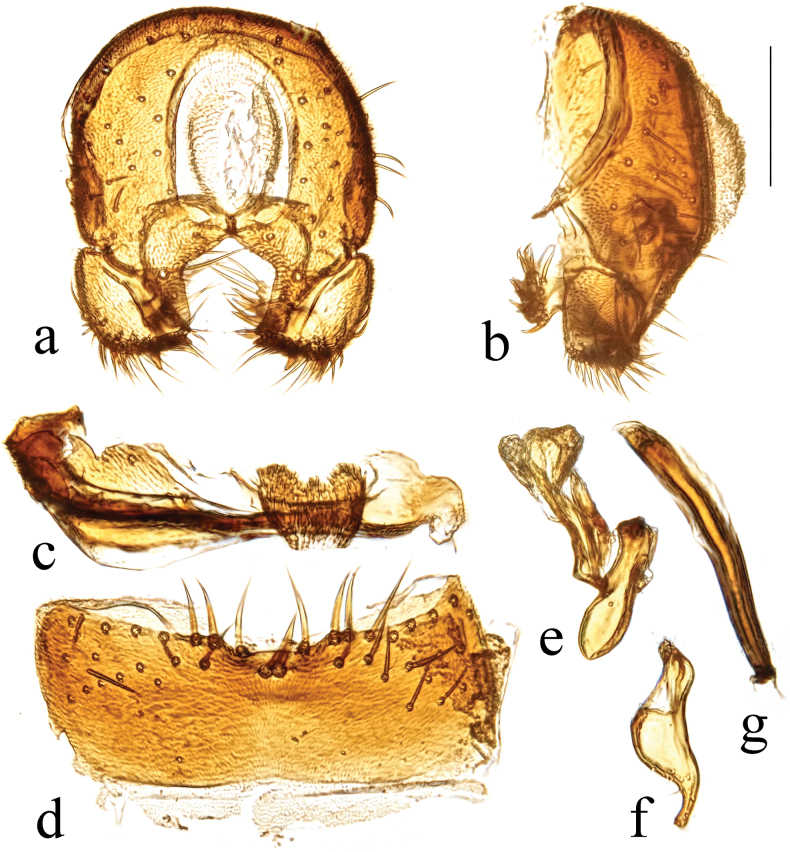
*T.paralongipexa* Hayashi, 1992 structures of male postabdomen. **a.** Terminalia posterior; **b.** Terminalia lateral; **c.** S_6_ ventral; **d.** S_5_ ventral; **e.** Distiphallus and basiphallus lateral; **f.** Postgonite ventral; **g.** Phallapodeme ventral. Scale bar: 0.1 mm.

#### Type locality.

Japan, Saitama, Iruma-gun, Moroyama.

#### Distribution.

China (Chongqing, Gansu, Guizhou, Hubei, Jiangxi, Jilin, Liaoning, Shaanxi, Xizang, Yunnan); Japan (Saitama).

#### Comments.

Previous Chinese records of this species are from Jiangxi, Jilin, Liaoning, and Shaanxi (Su and Liu 2009; Su 2011; Dong et al. 2020). Here we present new records from Chongqing, Gansu, Guizhou, Hubei, Tibet, and Yunnan.

### ﻿ Terrilimosina parasmetanai

Taxon classificationAnimaliaDipteraSphaeroceridae

Su & Liu, 2009

0BD9AF8C-DFAC-5555-B574-430DD2BEA253

Figs 1c , 24 , 25

Terrilimosina parasmetanai Su & Liu, 2009: 55. - Su 2011: 122–123, 219 [redescription, illustr.]; Dong et al. 2020: 431 [Species Catalog of China].

#### Material examined.

China: - **Beijing**: • 1 ♂; Mentougou, Xiaolongmen National Forest Park; 1330 m, 13.vi.2023; Yuetian Gao. - **Gansu**: • 2 ♂, 7 ♀; Linxia Hui Autonomous Prefecture, Hezheng County, Songmingyan Scenic Area; 2452 m, 11.viii.2023; Ding Yang. - **Hebei**: • 1 ♂; Shijiazhuang, Wuyuezhai, Qinvfeng; 6.vii.,2016; Xiao Zhang. - **Qinghai**: • 1 ♂; Menyuan, Laohugou; 3313 m, 26.vii.2023; Liang Wang. - **Shanxi**: • 1 ♂, 1 ♀; Lvliang, Pangquangou, Badaogou; 1930 m, 15.vii.2023; Leyou Zhang. - **Xizang**: • 1 ♂; Sejilashan, Xipo 1 hao; 3336 m, 30.vii.–23.viii.2023; Dawei Hong; (M). - **Yunnan**: • 4 ♂; Lushui, Pianmazhen; 3105 m, 12.viii.2023; Qicheng Yang. (all EMCAU).

#### Brief diagnosis of female.

**Figure 24.**
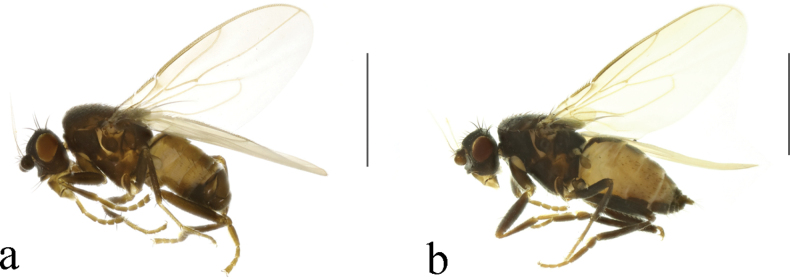
*T.parasmetanai* Su & Liu, 2009 habitus. **a.** Male lateral; **b.** Female lateral. Scale bars: 1.0 mm.

**Figure 25.**
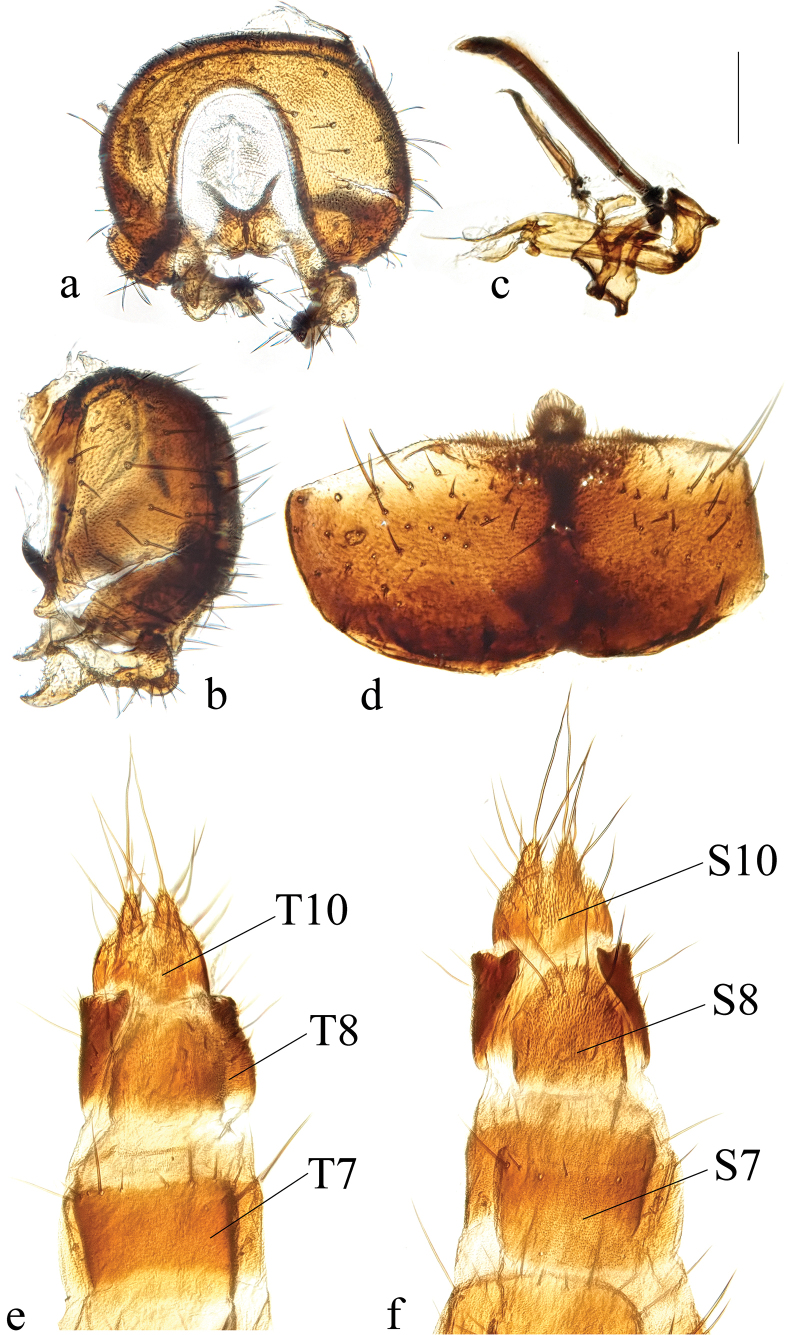
*T.parasmetanai* Su & Liu, 2009. **a–d.** Structures of male postabdomen: **a.** Terminalia posterior; **b.** Terminalia lateral; **c.** Phallus and associated structures lateral; **d.** S_5_ ventral; **e, f.** Structures of female postabdomen: **e.** Dorsal; **f.** Ventral. Scale bar: 0.1 mm.

The female (Figs 24b, 25e, f) of this species is newly associated. T_7_ rectangular, with two pairs long posterior setae and some small setae. T_8_ wide, extended laterally and tapered ventrally on each side. Epiproct with pair of setae. Hypoproct with narrow anteromedial process. Cercus with long and sinuate setae. Spermathecae (2+1) cylindrical, constricted medially, with narrow apical invagination.

#### Diagnosis.

Male S_5_ with a small densely setulose posteromedial process(Fig. 25d). Cercus short. Surstylus with a very small comb-like row of surstylar bristles ventrally (Fig. 25a–c).

#### Type locality.

China, Ningxia Province, Mt. Liu-p’an.

#### Distribution.

China (Beijing, Gansu, Hebei, Ningxia, Qinghai, Shaanxi, Tibet, Yunnan).

#### Comments.

Previous Chinese records of this species are from Ningxia (Su and Liu 2009; Su 2011; Dong et al. 2020). Here we present new records from Beijing, Gansu, Hebei, Qinghai, Shaanxi, Tibet, and Yunnan. Females of *Terrilimosinaparasmetanai* have four long posterior setae on S_8_ and two pairs of long setae on the hypoproct with narrow anteromedial process (Fig. 25e, f).

### ﻿ Terrilimosina schmitzi

Taxon classificationAnimaliaDipteraSphaeroceridae

(Duda, 1918)

A0291AC1-EF1F-5D66-A64D-B9A2122EA6F2

Figs 1g , 26 , 27

Limosina (Scotophilella) schmitzi Duda, 1918: 111.
Terrilimosina schmitzi . - Roháček et al. 2001: 273 [World Catalog of Sphaeroceridae]; Su and Liu 2009: 52 [illustr.]; Su 2011:124–125, 220 [redescription, illustr.]; Dong et al. 2020: 431 [Species Catalog of China].

#### Material examined.

China: - **Beijing**: • 1 ♂, Miyunxian, Huayuancun; 672 m, 3.vi.2018; Ding Yang. - **Hebei**: • 1 ♂; Zhangjiakou, Heilongshanlinchang, Beigou; 1421 m, 7.vii.2016; Wenmin Xiao. - **Jilin**: • 4 ♂, 3 ♀; Wangqing, Cilaoyagou; 500 m, 3.vii.2023; Yuetian Gao. (all EMCAU).

#### Diagnosis.

Male S_5_ with some setae and membranous posteromedial area (Fig. 27d). Surstylus broad, with some long setae at posterior corner and a short posteromedial process (Fig. 27b).

**Figure 26.**
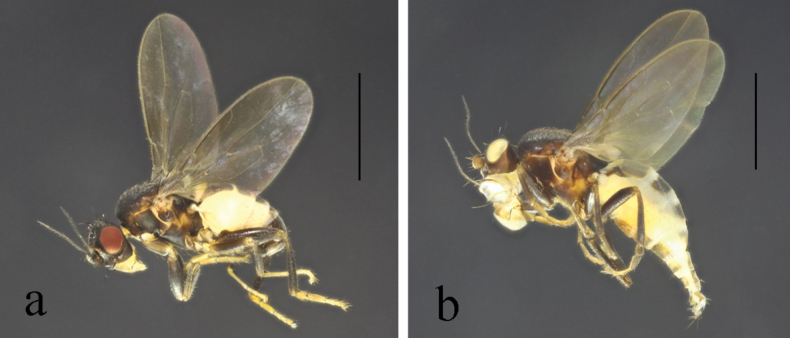
*T.schmitzi* (Duda, 1918) habitus. **a.** Male lateral; **b.** Female lateral. Scale bars: 1.0 mm.

**Figure 27.**
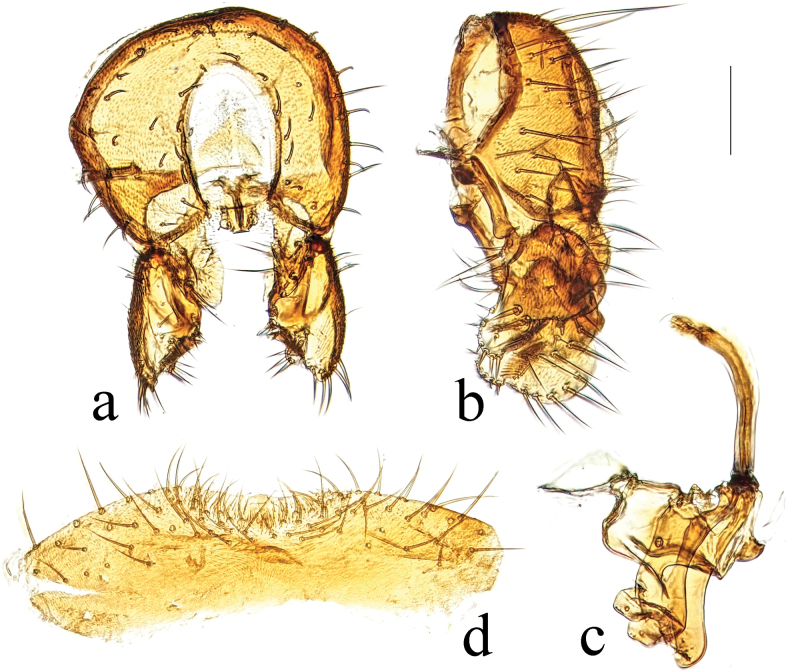
*T.schmitzi* (Duda, 1918) structures of male postabdomen. **a.** Terminalia posterior; **b.** Terminalia lateral; **c.** Phallus and associated structures lateral; **d.** S_5_ ventral. Scale bar: 0.1 mm.

#### Type locality.

Germany, Südharz, Ilfeld.

#### Distribution.

Austria; Belgium; Canada; China (Beijing, Hebei, Jilin, Liaoning); Czech Republic; Denmark; Finland; France; Germany; Great Britain; Hungary; Iceland; Italy; Latvia; Mongolia; Netherlands; North Korea; Norway; Poland; Romania; Russia; Slovakia; Spain; Sweden; Switzerland.

#### Comments.

Previous Chinese records of this species are from Jilin and Liaoning (Su and Liu 2009; Su 2011; Dong et al. 2020). Here we present new records from Beijing and Hebei.

### ﻿ Terrilimosina unio

Taxon classificationAnimaliaDipteraSphaeroceridae

Marshall, 1987

39543250-68D7-500A-BA37-2C7B55BC15BA

Figs 1m , 28 , 29

Terrilimosina unio Marshall, 1987: 508. - Roháček et al. 2001: 272 [World Catalog of Sphaeroceridae].

#### Material examined.

China: **Beijing**: • 1 ♂; Haidian, Jiufeng; 20.vi.2018, Jiale Zhou. **Guangxi**: • 1 ♂; Tonglezhen, Shenmutiankeng; 1200 m, 19.iv.2024; Wenqiang Cao. **Xizang**: • 13 ♂, 25 ♀; Motuo, 80K; 2125 m, 20.viii.2020; Qicheng Yang. **Yunnan**: • 2 ♂; Lushui, Pianmadaolu; 2284 m, 13.viii.2023; Qicheng Yang. (all EMCAU).

#### Diagnosis.

Syntergite 1+2 with weakly sclerotized anteromedial patch. Sternite 5 with two small patches of short but stout posterior bristles (Fig. 29d). Surstylus with two very short, blunt bristles at the apex of the posteroventral lobe (Fig. 29a–c).

**Figure 28.**
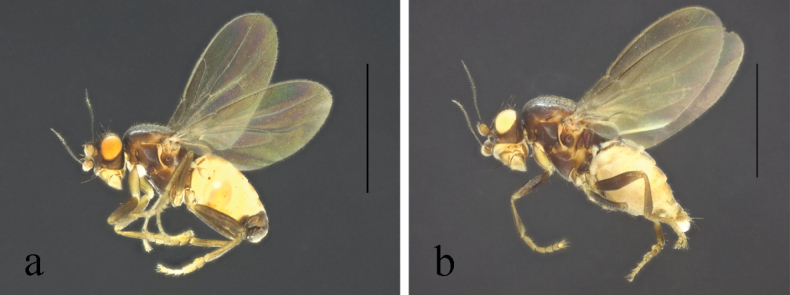
*T.unio* Marshall, 1987 habitus. **a.** Male lateral; **b.** Female lateral. Scale bars: 1.0 mm.

**Figure 29.**
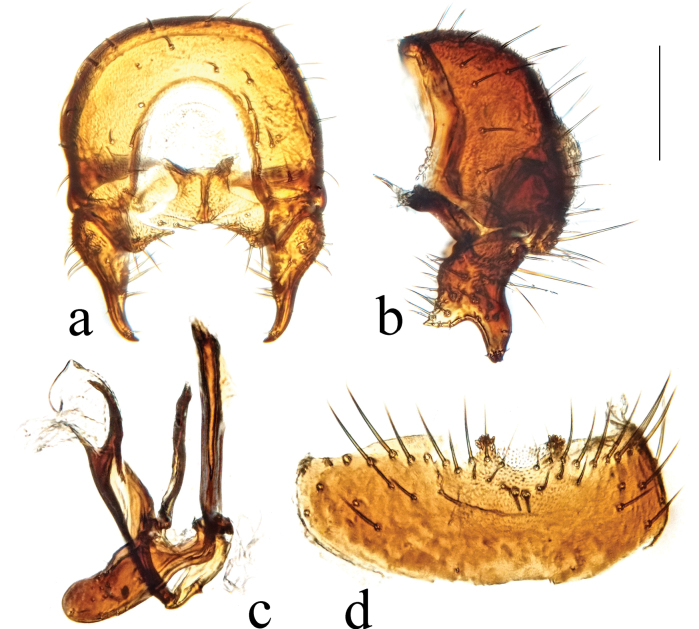
*T.unio* Marshall, 1987 structures of male postabdomen. **a.** Terminalia posterior; **b.** Terminalia lateral; **c.** Phallus and associated structures lateral; **d.** S_5_ ventral. Scale bar: 0.1 mm.

#### Type locality.

Nepal, Taplejung Distr., river banks below Tamrang Bridge [5500 ft].

#### Distribution.

China (Beijing, Xizang, Yunnan); Indonesia; Nepal.

#### Comments.

*Terrilimosinaunio* Marshall, newly recorded from China, resembles *T.brevipexa* Marshall, from which it differs in having a weakly sclerotized anteromedial patch on syntergite 1+2, two small patches of short but stout posterior bristles on S_5_ (Fig. 29d), and two very short, blunt bristles at the apex of the posteroventral lobe of the surstylus (Fig. 29a–c). *Terrilimosinabrevipexa* Marshall has a uniformly sclerotized syntergite 1+2, a patch of posteromedial setulae on S_5_ (Fig. 5d), and ~ 5 very short, blunt bristles at the apex of the posteroventral lobe of the surstylus (Fig. 5a–c).

## Supplementary Material

XML Treatment for Terrilimosina bicruris

XML Treatment for Terrilimosina brevipexa

XML Treatment for Terrilimosina capricornis

XML Treatment for Terrilimosina deemingi

XML Treatment for Terrilimosina dentata

XML Treatment for Terrilimosina digitata

XML Treatment for Terrilimosina longipexa

XML Treatment for Terrilimosina maoershanensis

XML Treatment for Terrilimosina nana

XML Treatment for Terrilimosina parabrevipexa

XML Treatment for Terrilimosina paralongipexa

XML Treatment for Terrilimosina parasmetanai

XML Treatment for Terrilimosina schmitzi

XML Treatment for Terrilimosina unio

## ﻿Additional information

### Author ORCIDs

Wenqiang Cao https://orcid.org/0009-0000-6484-1069

Hui Dong https://orcid.org/0009-0001-8917-5450

Ding Yang https://orcid.org/0000-0002-7685-3478

### Data availability

All of the data that support the findings of this study are available in the main text.
